# BRCA mutations lead to XIAP overexpression and sensitise ovarian cancer to inhibitor of apoptosis (IAP) family inhibitors

**DOI:** 10.1038/s41416-022-01823-5

**Published:** 2022-04-30

**Authors:** Mattia Cremona, Cassandra J. Vandenberg, Angela M. Farrelly, Stephen F. Madden, Clare Morgan, Roshni Kalachand, Jessica N. McAlpine, Sinead Toomey, David G. Huntsman, Liam Grogan, Oscar Breathnach, Patrick Morris, Mark S. Carey, Clare L. Scott, Bryan T. Hennessy

**Affiliations:** 1 Department of Molecular Medicine, Laboratory of Medical Oncology, Royal College of Surgeons in Ireland, Dublin, Ireland; 2 Cancer Biology and Stem Cells Division, Walter and Eliza Hall Institute of Medical Research, Parkville, VIC Australia; 3 Department of Medical Biology, University of Melbourne, Parkville, VIC Australia; 4 Data Science Centre, Royal College of Surgeons in Ireland, Dublin, Ireland; 5 Department of Obstetrics and Gynaecology, University of British Columbia, Vancouver, Canada; 6 BC Cancer, Vancouver, Canada; 7 Department of Pathology and Laboratory Medicine and Obstetrics and Gynaecology, University of British Columbia, Vancouver, Canada; 8 Department of Medical Oncology, Beaumont Hospital, Dublin, Ireland; 9 The Royal Women’s Hospital, Parkville, VIC Australia; 10 Department of Obstetrics and Gynaecology, Parkville, VIC Australia; 11 Peter MacCallum Cancer Centre, Parkville, VIC Australia

**Keywords:** Ovarian cancer, Chemotherapy

## Abstract

**Background:**

We tested the hypothesis that inhibitor of apoptosis family (IAP) proteins may be altered in *BRCA1*-mutated ovarian cancers and that could affect the sensitivity to IAP inhibitors.

**Methods:**

The levels of IAP proteins were evaluated in human cancers and cell lines. Cell lines were used to determine the effects of IAP inhibitors. The in vivo effects of treatments were evaluated in PDX mouse models.

**Results:**

Expression of X-linked inhibitor of apoptosis (XIAP) is increased in *BRCA1*-mutated cancers and high levels are associated with improved patient outcomes after platinum chemotherapy. XIAP overexpression is mediated by NF-kB activation and is associated with an optimisation of PARP. *BRCA1*-mutated cell lines are particularly sensitive to IAP inhibitors due to an inhibitory effect on PARP. Both a *BRCA1*-mutated cell line with acquired resistance to PARP inhibitors and one with restored *BRCA1* remain sensitive to IAP inhibitors. Treatment with IAP inhibitors restores the efficacy of PARP inhibition in these cell lines. The IAP inhibitor LCL161 alone and in combination with a PARP inhibitor, exhibited antitumour effects in PDX mouse models of resistant BRCA2 and 1-mutated ovarian cancer, respectively.

**Conclusion:**

A clinical trial may be justified to further investigate the utility of IAP inhibitors.

## Introduction

The *breast cancer 1, early onset (BRCA1)* germline and somatic mutation rates are ~11.5% and 7%, respectively, in ovarian cancer (OC) [1–4]. *BRCA1* mutations cause loss of BRCA1 protein function and, as a result, DNA repair by homologous recombination (HR) is defective [5]. This renders OC cells with *BRCA1* mutations specifically sensitive to cytotoxicity by platinum-based chemotherapy drugs [6], and by Poly (ADP-ribose) polymerase (PARP) inhibitors [6].

*BRCA1* also plays a role in other biological processes such as apoptosis [7]. Apoptosis plays a key role in cancer development and the anti-apoptotic inhibitor of apoptosis (IAP) family has a central role in this process [8]. The IAP family comprises 8 members, but only the X-linked inhibitor of apoptosis (XIAP) can directly inhibit caspases, the final effectors of apoptotic signalling [8]. IAP proteins have also been implicated in the control of non-apoptotic processes, including differentiation, cell motility, migration, invasion, and metastasis [9–11]. Elevated IAP protein levels (XIAP, cIAP1, cIAP2 in particular) are common in many cancer types including ovarian cancer and this is one mechanism that underlies the resistance of cancer cells to apoptosis [12]. Therefore, the inhibition of IAPs could increase the amount of apoptosis in cancer cells through caspase activation, which may of course be beneficial to cancer patients in the clinical setting [12]. Because of their ability to directly and indirectly regulate caspases, inhibitors of IAP family members have thus been developed as potential anticancer therapies [12].

The second mitochondria-derived activator of caspases (SMAC) mimetics are currently the most widely used class of IAP inhibitors and apoptosis-modulating drugs in cancer clinical trials [9]. SMAC is an endogenous factor [13] that antagonises IAP-mediated caspase inhibition [8] (i.e. an endogenous IAP inhibitor), and also induces proteasomal degradation of some members of the IAP family [14].

Because *BRCA1* plays a role in the regulation of apoptosis [15] and because *BRCA1*-mutated OCs are particularly sensitive to inhibition of PARP, a well-known caspase target, we tested the hypothesis that XIAP, cIAP1 or cIAP2 may be altered in *BRCA1*-mutated OCs and that this may have implications for the sensitivity of these OCs to SMAC mimetics.

## Methods

### Reverse-phase protein array (RPPA)

RPPA was performed as previously published by us [16] (more details in the Supplementary Data). The following antibodies were used: Parp-1#sc-7150 (Santa Cruz, TX, USA). Cleaved caspase-9 (Asp315) #9505, cleaved caspase-9 (Asp330) #9501, cleaved caspase-7 (Asp198) #9491 and XIAP#2042 (Cell Signaling, MA, USA).

### Cell-line panel

The following ovarian cancer cell lines were used: SNU-251, UWB1289, OVCAR8, OVCAR3, SKOV3, HOC1, OC316, MPSCI, PA-1, OCC1, PEO4, FUOV1, IGROV1, HEY, HIO-180, ES-2, A2780, DOV13 and UWB1289-BRCA1. HOC1 and IGROV1 were grown in DMEM (Invitrogen, CA, USA, #11995) with 10% FBS (Hyclone-GE Healthcare, UK, #sv30014.03). FUOV1 was grown in DMEM:F12 (Invitrogen, CA, USA, #11330) with 10% FBS. SNU-251 was grown in RPMI-1640 with 10% FBS with the addition of 10 μg/ml insulin (Invitrogen, CA, USA, #12585-014). UWB1289 and UWB1289-BRCA1 were grown in 50% RPMI-1640 with 10% FBS plus 50% MEGM (Lonza, CH, #cc-3150). The remainder of the cell lines were grown in RPMI-1640 plus 10% FBS. See the Supplementary Data for more detail about the CLs, the sources of the CLs and the procedures used for their authentication.

### Human ovarian cancer cohorts

The characteristics of cohort A has been already published by us [17]. Briefly, the samples were collected by the Gynecology Tumour Group at Vancouver General Hospital and the British Columbia Cancer Agency. The collection and study of these tissues were approved by the University of British Columbia Ethics Review Board (H14-02850/H19-02823). Tumour samples were collected in the operating room at the time of primary surgery and snap frozen within 30 minutes after collection in liquid nitrogen. Samples were processed in accordance with tissue bank guidelines and stored at −120 °C. The clinical data for the cohort B and C was obtained from publicly available data from the TCGA Research Network (http://cancergenome.nih.gov/) and from Yang et al. [18], respectively.

### Cytotoxicity-proliferation assays

Acid phosphatase assays were performed as previously published by us [19] (more details in Supplementary Data).

### Drugs

BV6 [20], cisplatin [21] and BMS-345541 [22] were obtained from Genentech (CA, USA) (under TMA), St. James’s University Hospital (IR) and from Sigma Aldrich (MO, USA), respectively. LCL161 [23] and olaparib were obtained from Selleck Chemicals (DE).

### siRNA transfection

Transfection of siRNA was performed using siPORT™ NeoFX™ Transfection agent (Ambion-Thermo Fisher, MA, USA) according to the manufacturer’s protocol. The following Ambion siRNAs were used: BRCA1-ID:S457,S458,S459 XIAP-ID:S1454,S1455 Negative Co-AM4611.

### Chromatin immunoprecipitation assay

ChIP assays were performed as previously published [24] (more details in Supplementary Data).

### DNA and RNA extraction

DNA and RNA were extracted using the Qiagen(DE) AllPrepDNA/RNA mini Kit™ by following the manufacturer’s protocol.

### *BRCA1/2* sequencing and the *BRCA1* promoter methylation assay

*BRCA1/2* mutation screening and the *BRCA1* promoter methylation assay were performed as previously published by us [19] (more details in Supplementary Data).

### Real-time quantitative PCR

Real-time quantitative PCR was performed with a 7500 FAST Real-time system (Thermo Fisher, MA, USA) using a TaqMan Universal Master Mix II with UNG according to the manufacturer’s protocol. The TaqMan primer sequences were as follows: XIAP #:4331182 ID:Hs00745222_s1, GAPDH #:4331182 ID:Hs02758991_g1.

### PARP1 activity assay

PARP1 Activity was evaluated as previously published [25]. In brief, the Trevigen (MD,USA) HT Universal 96-well PARP Assay Kit (#4677-096-K) was used according to the manufacturer’s protocol. In total, 20 μg of protein lysate from each sample was used and PARP1 activity in the treated samples was expressed as a percentage of PARP1 activity in the matched control-treated samples.

### Western blot

Western blot was performed as previously described by us [16]. Briefly, the gel image was acquired with a ChemiDOC MP (Biorad, CA, USA) and densitometry was performed using ImageJ software [26]. Protein intensity was subsequently normalised with the level of expression of GAPDH protein in the corresponding CLs. The following antibodies were used: GAPDH#sc-47724 and parp-1#sc-7150 (Santa Cruz, TX, USA)). XIAP#2042, NF-kB1 p105/p50#12540, NF-kB p65#8242 and NF-kB p65 (Ser536)#3033 (Cell Signaling, MA, USA).

### Evaluation of apoptosis

Apoptosis was evaluated using the Guava^®^ TUNEL Kit (#:4500-0121) (Millipore, MA, USA) by following the manufacturer’s protocol.

### Treatment of PDX

All mouse experiments were approved by the Walter and Eliza Hall Institute Animal Ethics Committee.

HGSOC PDX models #13, #56, #62 and #201 have been published previously [27, 28]. PDX model #931 was generated by subcutaneous transplantation of fresh patient tumour tissue fragments into 6-8 weeks old NOC/SCID/IL2Rγnull female mice (details in Supplementary Table 3). Mouse cohorts for treatment studies were produced by serial subcutaneous transplantation of PDX tumour fragments. From our previous studies 3–6 mice are sufficient to see clear and significant differences in response. Seven mice bearing tumours of 180–300 mm^3^ in volume were randomly assigned to each treatment. We did not use a randomisation tool. We fill across treatments groups equally as tumours reach treatment size. The researchers who assigned the treatments were not the same researcher who measured the tumours.

Cisplatin was administered by intraperitoneal injection at 4 mg/kg (in PBS) on days 1, 8 and 18; olaparib in 10% DMSO/10% 2-hydroxypropyl-β-cyclodextrin/PBS was administered at 50 or 100 mg/kg, by intraperitoneal injection daily (Monday–Friday) for 3 weeks; LCL161 was dissolved in 30% 0.1 N HCl/70% 100 mM sodium acetate buffer and administered by oral gavage at a dose of 100 mg/kg twice per week (Tuesday and Friday) for 3 weeks. The LCL161 vehicle was used as the vehicle control.

Tumours were measured twice per week with digital calipers and data entered directly into StudyLog (StudyLog Systems). The experimental endpoints were tumour volume of 700 mm^3^ or 120 day following the initiation of treatment. We excluded any mice that did not complete treatment due to weight loss or other humane endpoints.

The researchers who administered the treatments and measured tumours were not involved in the development of the project and were unaware of the project hypothesis. The data were analysed by another group of researchers. Tumour volume and Kaplan–Meier survival graphs were produce using SurvivalVolume v1.2 (https://github.com/genomematt/survivalvolume).

### Statistical analysis

The two-sided Student’s *t-*test (or Mann–Whitney’s test when appropriate) was used to compare differences between study groups, with a *P* value ≤0.05 considered statistically significant. The experiments were replicated three times. Before the analysis we test the population of samples for type of distribution, we estimate the variation within the data and we ensure that the variance between the groups was similar. The log-rank test (Mantel–Cox) was used to compare differences between the Kaplan–Meier survival curves. The Cox regression was used for investigating the effect of several variables on survival. All the analysis on patient survival was performed with MedCalc 16.2 (MedCalc software, BE). Graphs were created with Prism 5.03 (Graphpad, CA, USA).

## Results

### Level of XIAP is correlated with the *BRCA1*-mutational status of OC cell lines (CL)

**Fig. 1:**
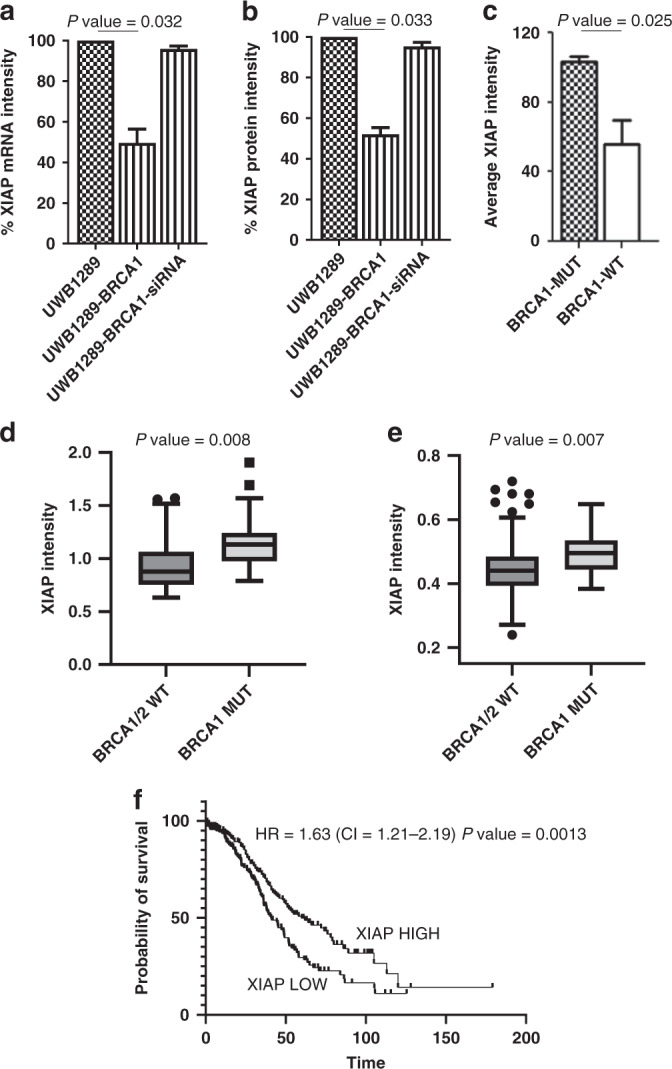
Correlation between XIAP and BRCA1-mutational status. **a** Level of expression of *XIAP* mRNA, evaluated by qPCR, in the *BRCA1*-mutated ovarian cancer (OC) cell line (CL) UWB1289, *BRCA1*-restored OC CL UWB1289-BRCA1 and UWB1289-BRCA1 after the silencing of wild-type *BRCA1*, using siRNA (siBRCA1) (*n* = 3). **b** The level of expression of XIAP protein, evaluated by western blot, in the *BRCA1*-mutated OC CL UWB1289, *BRCA1*-restored OC CL UWB1289-BRCA1 and in UWB1289-BRCA1 after the silencing of wild-type *BRCA1*, using siRNA (siBRCA1) (*n* = 3). **c** Comparison of the level of expression of XIAP protein, evaluated by western blot, in *BRCA1*-mutated OC CLs (BRCA1-MUT) and the *BRCA1/*BRCA2 wild-type OC CLs (BRCA1/2-WT) (*n* = 3). Effect size: Cohen’s *d* = (103–67.4)/38.300737 = 0.929486. **d** Evaluation by RPPA of the level of expression of XIAP protein in ovarian cancers (OCs) from 69 patients treated by primary surgery followed by platinum/taxane chemotherapy. XIAP levels were higher in OCs with *BRCA1* mutations (BRCA1-MUT) than in OCs with wild-type *BRCA1/2* (BRCA1/2-WT). Effect size: Cohen’s *d* = (1.174 − 0.949)/0.269438 = 0.835072. **e** In a separate group of 292 high-grade serous OCs, XIAP levels, as determined by RPPA, were also higher in OCs with *BRCA1* mutations (BRCA1-MUT) than in OCs with wild-type *BRCA1/2* (BRCA1/2-WT). Effect size: Cohen’s *d* = (0.4965 − 0.4408)/0.052942 = 1.0521. **f** In a group of 422 OCs from patients treated with surgery and adjuvant platinum-based chemotherapy, those patients with cancers expressing a level of XIAP protein higher than the median had a significantly improved overall survival. Note that in (**a**–**c**), XIAP protein or mRNA intensity in the CLs is calculated as a percentage of that in UWB1289 after normalisation with the level of expression of GAPDH protein or mRNA in the corresponding CLs. **d**, **e** XIAP protein intensity values in OCs were quantified by RPPA as described in “"Methods” and the values were visualised in the graphs (**d**, **e**) using Tukey boxplots.

We explored the possible correlation between IAP expression and *BRCA1* mutational status assessing the expression of *XIAP* by qPCR and western blot (WB) in both the *BRCA1*-mutated OC CL UWB1289 and the isogenic OC CL UWB1289-BRCA1 (which has wild-type *BRCA1* restored by transfection of a pcDNA3 plasmid-carrying wild-type *BRCA1* resulting in restoration of HR competence as shown in Supplementary Fig. 1). The levels of XIAP mRNA (Fig. 1a) and protein (Fig. 1b and Supplementary Fig. 2A) were higher in the *BRCA1*-mutated compared with the *BRCA1*-restored OC CL. To further explore this interaction, we silenced wild-type *BRCA1* in UWB1289-BRCA1 using siRNA to wild-type *BRCA1*. The silencing of *BRCA1* in UWB1289-BRCA1 led to an increase of XIAP mRNA and protein expression to levels comparable with those in the *BRCA1*-mutated CL UWB1289 (Fig. 1a, b and Supplementary Fig. 2D). These data suggest that, in this specific cell-line model, transcriptional changes underlie *BRCA1*-associated changes in XIAP protein levels.

In contrast to XIAP, we did not find the levels of the other IAP members to be associated with *BRCA* mutation status in the OC CLs (Supplementary Fig. 3A, B). We then evaluated the correlation between *BRCA1* mutation status and XIAP in a bigger panel of 17 OC CLs (Characteristics of cell lines in Supplementary Table 4). By *BRCA1/2* sequencing and methylation analysis, the panel was subdivided as follows: deleterious *BRCA1* mutants (SNU-251 and UWB1289), *BRCA1* methylated with reduced expression of *BRCA1* mRNA (OVCAR8) and 15 *BRCA1/BRCA2* wild-type CLs. There were no mutations or methylation of *BRCA2* in the cell-line panel. The level of expression of XIAP protein (as assessed by WB Supplementary Fig. 4A, B) was higher in the *BRCA1*-mutated OC CLs compared with the *BRCA1* wild-type OC CLs (Fig. 1c), consistent with our finding above.

The analysis was repeated on a sub-set of our panels comprising ten high-grade serous ovarian tumours. This smaller dataset shows the same trend with the level of expression of XIAP protein higher in the nine *BRCA1*-mutated OC CLs compared with the 1 *BRCA1* wild-type OC CL. Even in this case the effect size remains strongly positive (Supplementary Fig. 4C, D).

### Expression of XIAP in *BRCA1*-mutated vs *BRCA1* wild-type OCs from patients

We evaluated the level of protein expression of XIAP using Reverse Phase Protein Array (RPPA) in high-grade serous ovarian tumours from 69 patients treated by primary surgery followed by platinum/taxane chemotherapy (dataset A—Supplementary Table 1). *BRCA1/2*-mutation status was confirmed by sequencing of DNA extracted from tumours in all cases. There were 22 *BRCA1-*mutated tumours, 4 *BRCA2-*mutated tumours and 43 tumours had no mutations in either *BRCA1* or *BRCA2*. By t-test analysis we found that XIAP protein levels were higher in tumours with *BRCA1* mutations (Fig. 1d). The number of tumours with a BRCA2 mutation in the dataset was too low for a similar analysis. In a separate dataset (dataset B—Supplementary Table 1) of 292 patients with high-grade serous ovarian cancer (from OV_tcga_pan_can_atlas_2018) (with 18 *BRCA1*-mutated OCs, 12 *BRCA2*-mutated OCs and 262 tumours with no mutations in either *BRCA1* or *BRCA2*), XIAP protein expression by RPPA was also significantly higher in *BRCA1*-mutated versus *BRCA1/2-*wild-type OCs (Fig. 1e), thereby definitively confirming an association between increased XIAP protein expression and *BRCA1* mutation status in OC. In contrast, cIAP1 protein levels were not significantly different between *BRCA1*-mutated and *BRCA1/2-*wild-type human OCs (Supplementary Fig. 5A).

### Correlation of XIAP with patient outcomes in OC

Since XIAP levels are increased in *BRCA1*-mutated OCs, and since patient outcomes are better for those with *BRCA1*-mutated than *BRCA1* wild-type OCs because of higher platinum sensitivity [29], we hypothesised that high intratumoural expression of XIAP may therefore be associated with improved OC patient outcomes.

We therefore evaluated a possible correlation between the expressions of XIAP protein (evaluated by RPPA) in OC and patient outcomes in 422 patients (dataset B plus dataset C—Supplementary Table 1) who received adjuvant platinum-based chemotherapy after surgery. Those patients expressing a level of XIAP protein higher than the median had an improved overall survival (OS) (Fig. 1f).

This is consistent with the favourable effects of *BRCA1* mutations on the survival of OC patients after surgery and platinum-based chemotherapy that we and others have published previously [2]. XIAP remained a significant predictor of OS when clinical factors (age, stage) were included in a multivariable model. In contrast to XIAP, we did not find the intratumoural levels of cIAP1 to be associated with patient survival in OC (Supplementary Fig. 5B).

### XIAP overexpression in *BRCA1*-mutated OC CLs is mediated through NF-kB dependent mechanisms

**Fig. 2:**
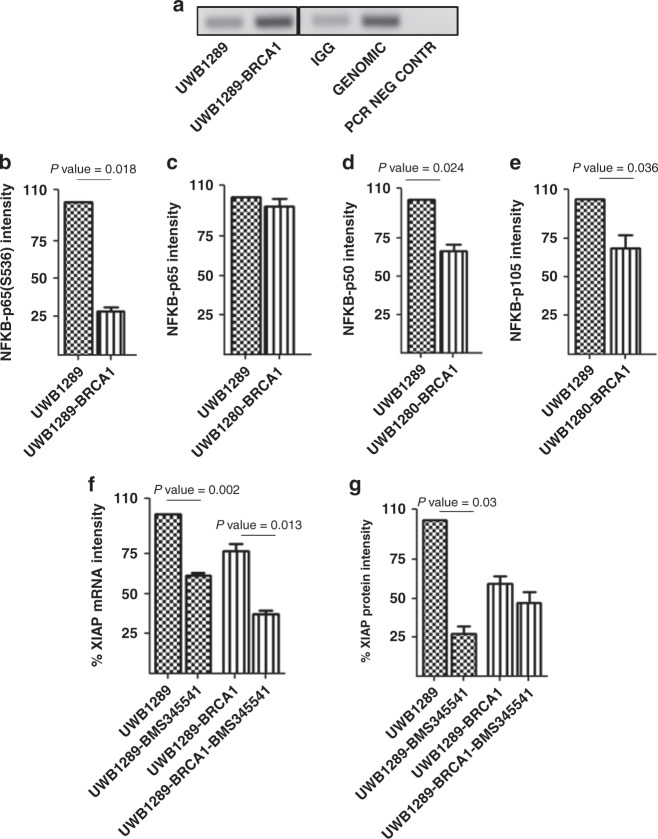
XIAP overexpression is mediated through NF-kB dependent mechanisms. **a** NF-kB p65 interacts with the promoter of *XIAP* in both the *BRCA1*-mutated UWB1289 and the isogenic UWB1289-BRCA1 cell lines (*n* = 2). **b**, **d**, **e** The level of phosphorylation of NF-kB –p65 at S536, NF-kB –p50 and NF-kB –p105, evaluated by western blot, were higher in the *BRCA1*-mutated ovarian cancer (OC CL) UWB1289 compared with the isogenic *BRCA1*-restored OC CL UWB1289-BRCA1 (*n* = 3). **c** The level of expression of NF-kB -p65 is similar in the UWB1289 and UWB1289-BRCA1 (*n* = 3). **f** Treatment with 5 µM BMS-345541 for 24 h decreased levels of XIAP mRNA in UWB1289 (BRCA1-MUT) and UWB1289-BRCA1 (BRCA1-restored) (*n* = 3). XIAP mRNA intensity is shown as a percentage of that in untreated UWB1289 after normalisation with the level of expression of GAPDH mRNA in the corresponding CLs. **g** Treatment with 5 µM of the inhibitor of NF-Kb pathway BMS-345541 for 24 h led to a decrease of XIAP protein expression only in the *BRCA1*-mutated OC CL UWB1289 (*n* = 3).

The different isoforms of nuclear factor kappa light-chain enhancer of activated B cells (NF-kB) have been shown interact with the *XIAP* promoter and to have a major role in the regulation of XIAP expression [24]. By co-immunoprecipitation, we confirmed that NF-kB p65 interacts with the promoter of *XIAP* in both the *BRCA1*-mutated UWB1289 and the isogenic UWB1289-BRCA1 OC CLs (Fig. 2a). Thus, changes in NF-kB may account for the increased expression of XIAP in *BRCA1*-mutated OC CLs. By western blotting, we found that the levels of the active forms of NF-kB (p65 phosphorylated at S563 and p50) and the precursor of p50 (p105) were significantly higher in *BRCA1*-mutated UWB1289 cells versus the *BRCA1*-restored OC CL UWB1289-BRCA1 (Fig. 2b, d, e and Supplementary Fig. 6). In contrast, we did not find the levels of the inactive form of p65 to be significantly different between the cell lines (Fig. 2c and Supplementary Fig. 6).

To confirm the specific role of NF-kB in regulating XIAP levels in *BRCA1*-mutated OC, we treated UWB1289 and isogenic UWB1289-BRCA1 cells with BMS-345541 (an allosteric site-binding inhibitor of IKK-2, thereby acting as an inhibitor of NF-kB-induced transcription [22]). BMS-345541 decreased XIAP mRNA (Fig. 2f) by ~50% in both OC CLs, with the level of XIAP protein decreased significantly only in the *BRCA1*-mutated CL UWB1289 (Fig. 2g).

We also explored if inhibition of NF-kB pathways with BMS-345441 might specifically inhibit the growth of *BRCA1*-mutated OC cell lines in our panel of 17 OC CLs. The BMS-345541 IC_50_s in the cell-line panel are shown in Supplementary Fig. 7A. The sensitivity of *BRCA1*-mutated OC CLs to the drug is increased in comparison with *BRCA1* wild-type OC cell lines (Supplementary Fig. 7B).

**Fig. 3:**
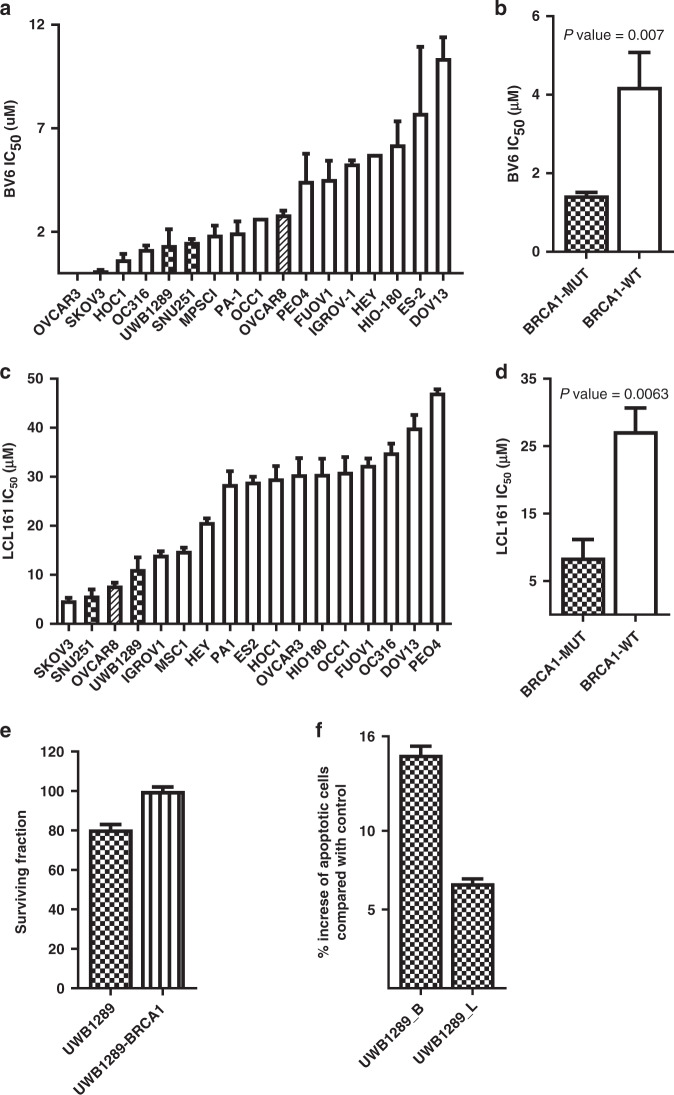
Sensitivity of ovarian cancer cell lines to IAP inhibitors. **a** Cytotoxicity of the IAP inhibitor BV6 in a panel of 17 ovarian cancer (OC) cell lines (CLs). **b** *BRCA1*-mutated OC CLs (BRCA1-MUT) are on average more sensitive to BV6 than *BRCA1* wild-type (BRCA1-WT) OC CLs. **c** Cytotoxicity of the IAP inhibitor LCL161 in a panel of 17 OC CLs. **d** *BRCA1*-mutated OC CLs are on average more sensitive to LCL161 than *BRCA1* wild-type OC CLs. **e** After the silencing of *XIAP*, using 25 nM siRNA (siXIAP), the proliferation of the *BRCA1*-mutated OC CL UWB1289 is downregulated in comparison to the *BRCA1*-restored OC CL UWB1289-BRCA1. These data were normalised to the proliferation of the same CLs after treatment with a control scrambled siRNA. **f** Evaluation of the level of apoptosis after treatment with BV6 (_B) and LCL161 (_L). Apoptosis is shown as a percentage increase of that in corresponding control-untreated CLs (*n* = 3). Note: *BRCA1*-mutated OC CLs are highlighted with a pattern, the *BRCA1*-methylated OC CL is highlighted with oblique banding, the B*RCA1*-restored OC CL is highlighted with horizontal banding and BRCA1 wild-type OC CLs are highlighted in white in the bar graphs.

Combined, the data in Figs. 1a, b and 3f, g suggest that XIAP overexpression in *BRCA1*-mutated OC cells is mediated through transcriptional and post-transcriptional mechanisms. Figure 3a tells us that NF-kB p65 interacts with the *XIAP* promoter and Fig. 3b–e imply that NF-kB activation but not expression levels are specifically high in *BRCA1*-mutated OC cells. The data in Fig. 3f, g suggest that NF-kB inhibition decreases XIAP mRNA and protein levels, the latter specifically in *BRCA1*-mutated OC cells through transcriptional and, more specifically post-transcriptional mechanisms.

NF-kB activation can be the result of TNFα, akt activation or reactive oxygen species (ROS) [30–32]. However, we found no differences between UWB1289 and UWB1289-BRCA restored in terms of effects of Infliximab (TNFα antibody), akt phosphorylation (at T308 or S473) or peroxide-induces ROS levels (Supplementary Fig. 8A–C).

### Sensitivity of OC cell lines to IAP inhibitors (IAPi) (SMAC mimetics)

Because XIAP levels are higher in *BRCA1*-mutated OCs, we explored whether XIAP inhibition might specifically inhibit the growth of *BRCA1*-deficient OC cell lines. We used the IAP inhibitors (IAPis) BV6 and LCL161 [33] in the panel of OC CLs.

The individual IC_50_s of BV6 in the cell-line panel are shown in Fig. 3a and Supplementary Table 2. *BRCA1*-mutated OC CLs are on average significantly more sensitive to BV6 (Fig. 3b) than *BRCA1* wild-type OC CLs, just as they are well known to be more sensitive to cisplatin (Supplementary Fig. 9A) and PARP inhibitors (Supplementary Fig. 9B).

To further confirm the effect of IAP inhibitors, we treated the 17 OC CLs in the panel with the alternative IAPi LCL161 (Fig. 3c and Supplementary Table 2). As with BV6, *BRCA1*-mutated OC CLs were also on average significantly more sensitive to LCL161 than *BRCA1* wild-type OC CLs (Fig. 3d).

We next evaluated the effect of *XIAP* silencing on the proliferation of the *BRCA1*-mutated OC CL UWB1289 and of the *BRCA1*-restored OC CL UWB1289-BRCA1. Treatment with *XIAP* siRNA led to a decrease of XIAP protein expression in both UWB1289 and UWB1289-BRCA1 in comparison with control-treated cells (Supplementary Fig. 9C). The proliferation of the *BRCA1*-mutated OC cell line was significantly downregulated by XIAP siRNA in contrast to the *BRCA1*-restored OC cell line (Fig. 3e). This is consistent with the effect of LCL161 and BV6 on the proliferation of OC cell lines shown above.

The induction of apoptosis after treatment with BV6, LCL161 was evaluated in the *BRCA1*-mutated OC cell line UWB1289. Treatment with BV6 and LCL161 induced significantly more apoptosis in the *BRCA1*-mutated OC cell line compared with the untreated control (Fig. 3f).

These data suggest that sensitivity to XIAP inhibitors is increased in *BRCA1*-mutated OC cell lines compared with *BRCA1* wild-type OC cell lines.

### IAPis act functionally as PARPis in OC cell lines

Because *BRCA1*-mutated OCs are specifically sensitive to PARP inhibition [6], just as is the case we have found with IAP inhibition, we hypothesised that IAPi such as BV6 and LCL161 may indirectly inhibit PARP given that PARP expression and cleavage are regulated by the caspases that are inhibited by XIAP.

**Fig. 4:**
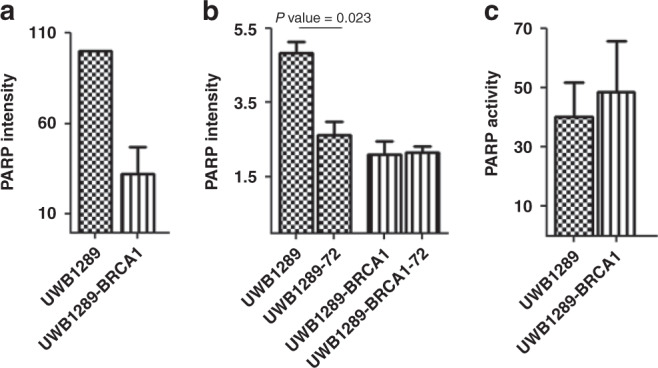
Effect of IAP inhibitor on PARP. **a** The level of expression of PARP protein, evaluated by western blot, was higher in the *BRCA1*-mutated ovarian cancer cell line (OC CL) UWB1289 compared with the *BRCA1*-restored OC CL UWB1289-BRCA1 (*n* = 3). **b** After 72 h of treatment with BV6 (−72), the level of PARP protein in UWB1289 is comparable with the PARP level in matched control-treated UWB1289-BRCA1. In contrast, the level of PARP in UWB1289-BRCA1 is not decreased by BV6 treatment. The PARP intensity values in the OCs were quantified by RPPA as reported in ''Methods''. **c** Treatment with BV6 impairs PARP activity in both cell lines. PARP activity is shown as a percentage of that in control-treated CLs (*n* = 3).

Previously published data have shown that PARP protein levels are increased in OCs with BRCA1 loss [34]. To confirm this possible correlation, we evaluated the level of PARP protein expression in UWB1289 and UWB1289-BRCA1, and found that it was indeed higher in UWB1289 (Fig. 4a) just as is the case with XIAP (Fig. 1b).

In addition, as might be expected, the level of cleavage (activation) of caspases 7 and 9, both targets of XIAP that can cleave and degrade PARP, was significantly lower in UWB1289 than in UWB1289-BRCA1 (Supplementary Fig. 10A–C).

We then treated the *BRCA1*-mutated and the *BRCA1-*restored isogenic OC cell lines with 0.5 μM BV6 for 72 h, a concentration that was associated with ≤20% inhibition of proliferation (Supplementary Fig. 11A). As expected, this treatment with the SMAC mimetic led to a decrease in IAP protein levels in both the CLs (Supplementary Fig. 11B). However, only in the *BRCA*1-mutated OC cell line did it lead to a decrease in total PARP protein levels and this decrease was to approximately the same level of PARP protein expression seen in *BRCA1* wild-type OC cell lines (Fig. 4b). BV6 treatment also led to decreased PARP activity in all OC cell lines tested (Fig. 4c).

These data confirm that SMAC mimetics IAPi lead to degradation and decreased levels of PARP protein specifically in *BRCA1*-mutated OC cell lines in addition to decreasing PARP activity in all cell lines. These data also suggest that XIAP overexpression in *BRCA1*-mutated OCs may facilitate reduced caspase cleavage (activity) and increased PARP expression and activity, the latter being a possible compensatory mechanism for HR deficiency.

### *BRCA1*-mutated ovarian cancers cell lines with acquired resistance to PARPis remain sensitive to IAPis

**Fig. 5:**
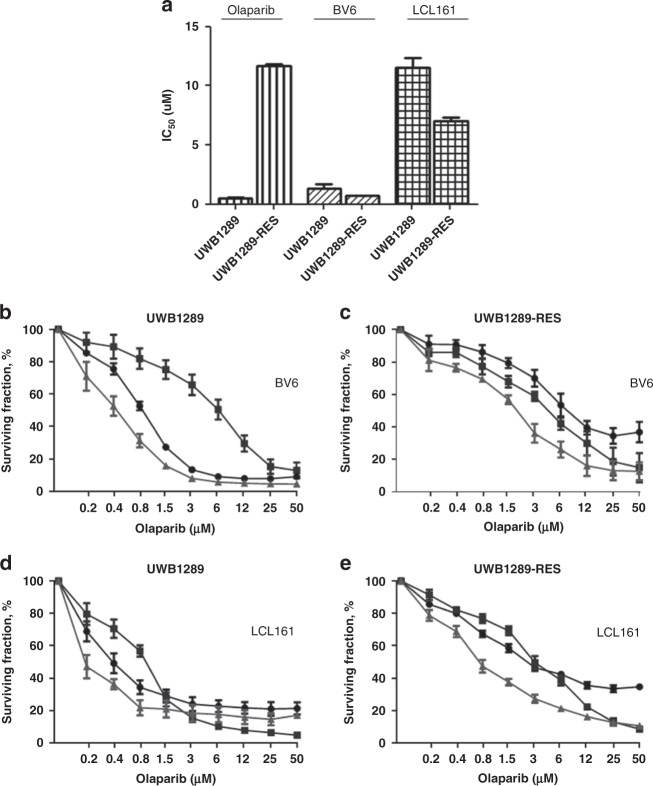
Effect of IAP inhibitors on an ovarian cancer cell line with acquired resistance to PARP inhibitor. **a** The cell line UWB1289-RES has acquired resistance to olaparib than the parental cell line. However UWB1289-olap-res remained sensitive to treatment with BV6 and LCL161 with no significant increase in IC_50_ (*n* = 3). **b**, **c** Efficacy of olaparib (PARPi) (-o-), BV6 (IAPi) (-□-) and a combination of olaparib and BV6 (-∆-) in the parental ovarian cancer cell line UWB1289 and UWB1289-olap-res with acquired resistance to olaparib. Error bars are representative of standard deviations across triplicate independent experiments. The ratio of olaparib:BV6 in this assay was fixed at 6.66:1. **d**, **e** Efficacy of olaparib (PARPi) (-o-), LCL161 (IAPi) (-□-) and a combination of olaparib and LCL161 (-∆-) in the parental ovarian cancer cell line UWB1289 and UWB1289-olap-res with acquired resistance to olaparib. Error bars are representative of standard deviations across triplicate independent experiments. The ratio of olaparib:LCL161 in this assay was fixed at 0.8:1.

*BRCA1*-mutated ovarian cancers develop resistance to PARPis relatively quickly, commonly through restoration of HR proficiency [35]. We developed a model of resistance to olaparib exposing UWB1289 to a prolonged treatment with olaparib. This second cell line (UWB1289-olap-res) was still *BRCA1*-mutated, however HR proficiency was restored, as shown in Supplementary Fig. 12. UWB1289-RES was significantly more resistant to olaparib than UWB1289. However UWB1289-RES remained sensitive to treatment with BV6 and LCL161, with no significant increase in IC_50_s in comparison with parental UWB1289 (Fig. 5a and Table 1A).

**Table 1:** (A, B) Values for the half maximal inhibitory concentration (IC_50_) and combination index (CI).

(A)									
	IC_50_	IC_50_	IC_50_	Combination					
	BV6 (µM)	Olaparib (µM)	Olaparib + BV6 (µM)	Index*					
UWB1289	1.36 ± 0.77	0.5 ± 0.1	0.3 ± 0.1	1.1 ± 0.05					
UWB1289-RES	0.7 ± 0.1	11.7 ± 0.25	1.88 ± 0.4	0.6 ± 0.03					
**(B)**									
	**IC**_**50**_ **LCL161 (µM)**	**IC**_**50**_ **Olaparib (µM)**	**IC**_**50**_ **Olaparib** + **LCL161 (µM)**	**Combination** Index*					
UWB1289	11.7 ± .2.7	0.5 ± 0.1	4.9 ± 0.7	1.12 ± 0.03					
UWB1289-RES	7 ± .0.5	11.7 ± 0.25	5.2 ± 0.3	0.76 ± 0.19					
**(C)**									
**PDX model**	**Vehicle**	**Olaparib**	**Olaparib 100** **mg/kg**	**LCL161**	**LCL161** + **Olaparib**	**LCL161** + **Olaparib 100** **mg/kg**			
#56	71	43	78	78	92	>120			
#62	18	36		22	36				
#13	60	64		50	57				
#931	25	32		43	36				
#201	22	43		32	39				
**(D)**									
**PDX model**	**Vehicle vs Olaparib**	**Vehicle vs LCL161**	**Olaparib vs LCL161**	**Vehicle vs** **LCL161** + **Olaparib**	**Olaparib vs** **LCL161** + **Olaparib**	**LCL161 vs** **LCL161** + **Olaparib**	**Vehicle vs** **LCL161** + **Olaparib 100** **mg/kg**	**Olaparib 100** **mg/kg vs** **LCL161** + **Olaparib 100** **mg/kg**	**LCL161 vs** **LCL161** + **Olaparib 100** **mg/kg**
#56	0.3893	0.8643	0.2308	0.3881	0.0482*	0.3793	0.077	0.069	0.0204*
#62	0.0271*	0.4904	0.0929	0.0024**	0.7055	0.0070**			
#13	0.4251	0.9893	0.4989	0.9676	0.5502	0.7305			
#931	0.0724	0.0070**	0.1374	0.0040**	0.5751	0.1332			
#201	0.2975	0.2233	0.1055	0.0926	0.8398	0.1241			

The IC_50_s and CIs (using the Chou–Talalay method) have been calculated for the PARP inhibitor olaparib and the IAP inhibitors BV6 and LCL161 in the *BRCA1*-mutated ovarian cancer cell line UWB1289 and the *BRCA1*-mutated ovarian cancer cell line with acquired resistance to olaparib UWB1289-RES.

(C) Median Time to Harvest (TTH) in days for HGSOC PDX models following treatment. Olaparib 50 mg/kg, unless otherwise indicated; LCL161 100 mg/kg.

(D) Comparison of response of HGSOC PDX models to treatment with olaparib (50 mg/kg), LCL161 (100 mg/kg) or combination treatment. *P* values were determined with the log-rank test, * indicates significance.

### Treatment with IAPis restores sensitivity to PARPis in *BRCA1*-mutated ovarian cancers cell lines with acquired resistance

Combinations of olaparib with the IAPis BV6 and LCL161 enhance growth inhibition relative to testing either drug alone in the olaparib resistant cell line UWB1289-RES (Fig. 5b–e and Table 1B). Moreover, the IAP inhibitors restore the effectiveness of the treatment with olaparib significantly decreases the IC_50_ compared with the treatment with an olaparib like a single agent.

The *BRCA1*-mutated OC cell line with acquired resistance to olaparib (UWB1289-RES) shows a deregulation of DNA repair by HR after exposure to IAPi with a significant accumulation of unrepaired DNA double-strand breaks (Supplementary Fig. 12). This may explain the synergy between IAPi and olaparib noted above.

### Sensitivity of in vivo PDX models to IAP inhibitor LCL161

**Fig. 6:**
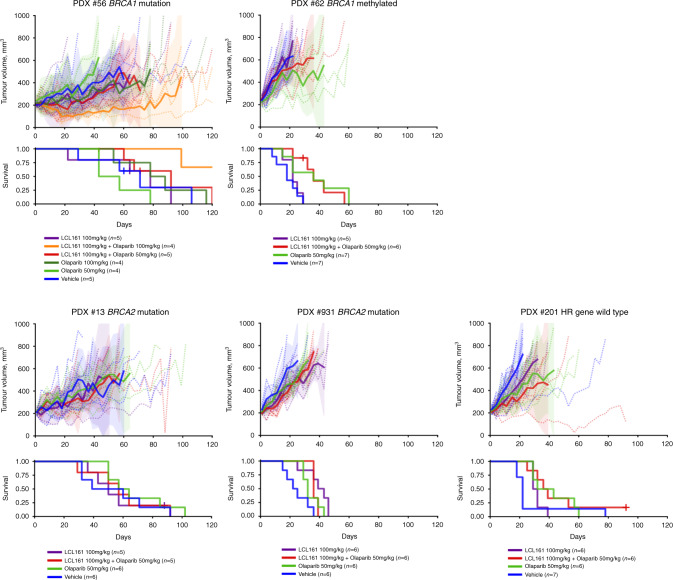
Sensitivity of in vivo PDX models to LCL161. Changes in tumour volumes and survival curve for PDX models after treatment with LCL161 and olaparib as single agents and in combination.

Five HGSOC PDX models were selected for in vivo treatment studies based on *BRCA1/2* status and in vivo cisplatin response: one BRCA1 mutated, one BRCA1 homozygously methylated, two BRCA2 mutated and one BRCA1/2-WT (Fig. 6). To assess IAPi sensitivity in vivo, LCL161 was delivered by oral gavage twice weekly for 3 weeks at the dose of 100 mg/kg. PDX#931 (*BRCA2* mutated, platinum-refractory), demonstrated delayed progression of disease in response to single agent LCL161 in vivo (Fig. 6 and Table 1C), median survival 43 days for LCL161- vs vehicle-treated mice 25 days (*P* = 0.0070).

To evaluate the effect of co-treatment with an IAPi and PARPi, mice were treated with LCL161 (same conditions as above) and olaparib: 5 days a week (Monday to Friday) for 3 weeks at the dose of 50 mg/kg. The *BRCA1-*mutated model, PDX #56, displayed a mixed response to the LCL161/olaparib combination with two out of five tumours showing stabilisation. As 100 mg/kg twice weekly is the maximum tolerated dose for LCL161, we increased olaparib from 50 mg/kg to 100 mg/kg and observed reduction in tumour volume in all four mice treated, maintained out to 60 days (median survival is >120 days for LCL161 + Olaparib 100 mg/kg vs 71 days for vehicle-treated mice (*P* = 0.0378) Table 1D).

For the BRCA2-mutated PDX #13, known to contain efflux mechanisms, we did not observe any effect of treatments; however, for the other three PDX models small improvements might suggest that treatment with the higher dose regimen could be worthwhile (Table 1C).

## Discussion

Herein, we demonstrate that *BRCA1*-mutated OCs overexpress XIAP protein and that *BRCA1*-mutated OC cell lines are specifically sensitive to growth inhibition by IAPi, as a result of indirect downregulation and inhibition of PARP by these drugs. *BRCA*-mutated OC with acquired resistance to PARPi also remains sensitive to IAPi. Moreover, we found that combining IAPi with the PARPi olaparib may result in synergistic antitumour effects relative to testing either drug alone in some *BRCA*-mutated OC with acquired resistance to PARPi. It should be noted that IAP inhibitors inhibit other IAP family members in addition to XIAP, in particular cIAP1. However, in contrast to XIAP, we did not find the levels of these IAPs to be associated with *BRCA* mutation status or patient outcomes in OC.

Overexpression of XIAP compared with matched normal tissues has been reported in different cancer types, including OC [36]. The results of our study show a stratification in the level of expression of XIAP protein within OC by *BRCA1* mutational status. XIAP protein is expressed at higher levels in *BRCA1*-mutated compared with *BRCA1* wild-type high-grade serous OCs and OC cell lines. Further, the inhibition of NF-kB, known to be one of the main regulators of *XIAP* transcription, leads to an ~50% decrease in XIAP mRNA levels in both *BRCA1*-mutated and *BRCA1*-restored UWB1289 cells but to a specific (80%) decrease in XIAP protein levels in only parental *BRCA1*-mutated UWB1289 cells. Therefore, our data suggest that NF-kB-regulated post-transcriptional processes are likely involved in the specific upregulation of XIAP protein in *BRCA1*-mutated OC and we are exploring this mechanism further at present.

Previously the correlation between XIAP expression in various cancers and patient prognosis has been evaluated and a high level of XIAP has been found to correlate with a good or poor prognosis depending on cancer type [37–40]. Our data show a favourable impact of high XIAP expression on the outcome of platinum-treated high-grade serous OC patients. Our results are derived from a combined analysis of two large datasets. Another study reported high expression of XIAP to correlate with a poor prognosis after platinum treatment in clear cell OC [41]. The discrepancy between this study and ours may be explained by differences between clear cell OCs and serous OCs (the latter being the main focus of our study) in terms of responsiveness to platinum treatment and differences in the incidence of *BRCA1* mutations. Our data suggest that high XIAP protein expression in high-grade serous OC could be a marker of *BRCA1* mutations and this may explain at least in part why high XIAP expression in OC is associated with improved patient outcomes after platinum chemotherapy.

IAP antagonists have demonstrated antitumour activity in several cancer xenograft models [9] and LCL161 is being evaluated in cancer clinical trials at present [42]. The results of our study show that IAP inhibitors specifically inhibit the growth of *BRCA1*-mutated OC cell lines. This is likely to be, at least partly, because IAP inhibitors indirectly downregulate and inhibit PARP. *BRCA1*-mutated OCs have been previously shown to be specifically sensitive to PARP inhibitors [43].

We found that PARP levels are higher in a *BRCA1*-mutated compared with the isogenic *BRCA1*-restored OC cell line (UWB1289-BRCA1). This result is consistent with previous studies [34]. Since HR-deficient *BRCA1*-mutated OCs have higher sensitivity to DNA damaging agents such as cisplatin, Wysham et al. suggested that PARP upregulation may be a compensatory mechanism for HR deficiency [34]. Our data support this hypothesis. In fact, *BRCA1*-mutated (and HR-deficient) OC cells rely on PARP for DNA repair and survival and our results suggest that high XIAP expression may function to maximise PARP levels in these cancer cells, since XIAP inhibits the caspases that cleave and degrade PARP.

The likely role of XIAP in maintaining high PARP levels in *BRCA1*-mutated OC cell lines is also supported by the effect of treatment of OC cell lines with IAPi. After exposure to two IAPi, the level of PARP protein in *BRCA*1-mutated OC cells decreases to approximately the baseline level of PARP expression seen in *BRCA1* wild-type OC cells, likely due to activation of caspases. Our data also show that there is inhibition of PARP activity by IAPi in all OC cell lines. This mechanism underlying this observation requires further study, but it could possibly also be explained by the activation of caspases. Since IAP inhibitors cause degradation and inhibition of PARP, this likely explains the higher sensitivity of *BRCA1*-mutated and HR-deficient OC cell lines to these drugs that we have shown for the first time herein.

The restoration of wild-type BRCA1 and thus HR proficiency to the BRCA1-mutated OC cell line UWB1289 greatly increases resistance to the PARPi olaparib, as expected, but not to IAPi. Moreover, during co-treatment with IAPi, the effect of olaparib is restored in the cell line with acquired resistance to the PARPi possibly due to impairment of HR by the IAPi. This observation needs further study, but it suggests a potential role for IAP inhibitors in the treatment of PARP-resistant BRCA1-mutated OCs.

Further, there are patients with “BRCA mutated-like” ovarian carcinomas and impaired HR (i.e: with amplification/overexpression of EMSY [44, 45]; with structural variances/hypermethylation in BRCA1/2 [46, 47] and with mutation in genes involved in HR [5, 48]). These patients could potentially also benefit from treatment with IAP inhibitors although this requires further study to confirm.

Our PDX studies support efficacy in *BRCA1/2* mutant HGSOC models. The PARPi and IAPi combination treatment appears most effective in the BRCA1 mutant PDX model PDX #56, a model with resistance to olaparib, thus supporting our in vitro data. Other PDX models only achieve a trend towards stabilisation of disease at the lower dose combination regimen. It is intriguing that the model with the highest benefit from single agent treatment with the IAPi is the one with the highest resistance to treatment with PARPi. This result also supports our in vitro data in cell lines with resistance to treatment with PARPi.

In summary, we provide evidence herein that *BRCA*-mutated OCs are particularly sensitive to novel IAPi. The specific lethality of IAPi in *BRCA1*-mutated OC cell lines could be partly due to the indirect modulation of PARP levels by these inhibitors. We also demonstrate a correlation between *BRCA1* mutation status and the level of XIAP protein expression. XIAP may also be a good marker for responsiveness to DNA damaging drugs in OC.

Limitations of this study include the genetic heterogeneity of the ovarian tumour cell lines and human samples used, and the fact that the correlation between XIAP and BRCA status could affect the survival of patients after treatment with IAPs inhibitors. Further studies are required to better understand what role, if any, these factors have.

From our preliminary in vitro*/*in vivo study data herein, we believe an early phase clinical trial may be justified to further investigate the potential utility of IAPi alone and in combination with PARPi in the treatment of *BRCA*-mutated OCs with resistance to PARPi.

## Supplementary information

supplementary data

checklist

## Data Availability

The dataset generated and/or analysed during the current study are available from the corresponding author on reasonable request.
