# Supplementary material for: BRCA mutations lead to XIAP overexpression and sensitise ovarian cancer to inhibitor of apoptosis (IAP) family inhibitors
Source: Br J Cancer. 2022 Apr 30;127(3):488–99. doi: 10.1038/s41416-022-01823-5 (PMC9345958; doi:10.1038/s41416-022-01823-5)
Supplement: Supplementary file 1 — supplementary data [file 41416_2022_1823_MOESM1_ESM.docx]

**BRCA mutations lead to XIAP overexpression and sensitise ovarian cancer to inhibitor of apoptosis (IAP) family inhibitors**

Mattia Cremona, Cassandra J. Vandenberg, Angela M. Farrelly, Stephen F. Madden, Clare Morgan, Roshni Kalachand, Jessica N. McAlpine, Sinead Toomey, David G. Huntsman, Liam Grogan, Oscar Breathnach, Patrick Morris^6^, Mark S. Carey, Clare L. Scott and Bryan T. Hennessy

UWBI289-BRCA1

Olaparib 0.5uM

UWBI289

Olaparib 0.5uM


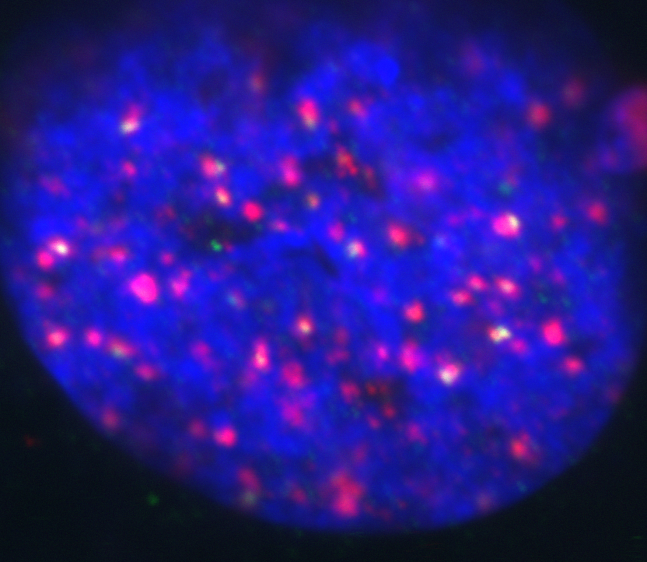

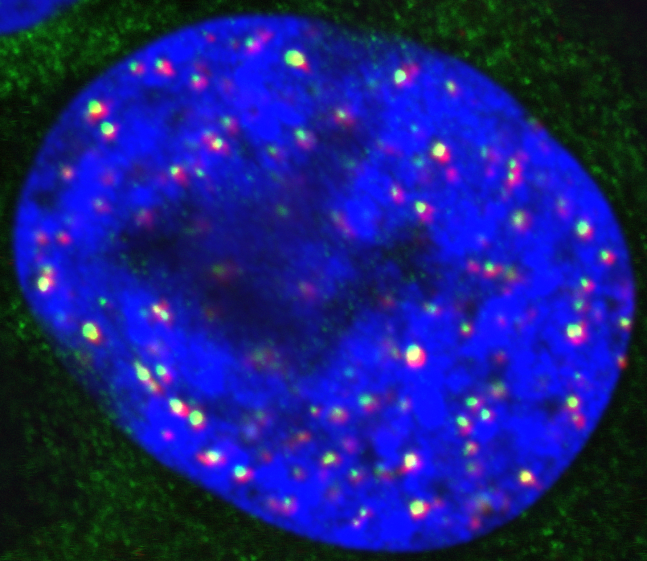


Fig.1 suppl. The transfection of a pcDNA3 plasmid carrying wild-type *BRCA1* in the *BRCA1*-mutated ovarian cancer cell line UWB1289 results in restoration of homologous recombination (HR) competence. HR competence was evaluated using an HR assay as reported by Mukhopadhyay *et al.* in Clin Cancer Res; 16(8) April 15, 2010. Briefly, the histone protein H2AX (red dot) interacts directly with chromatin creating a focus where proteins involved in DNA repair accumulate. Rad51 (green dot) is a crucial down-stream protein involved in HR repair, one of the last proteins interacting with the complex involved in HR repair. Quantification of H2AX-Rad51 complexes serves as a marker of HR function to distinguish between HR-proficient and HR-deficient cell lines.


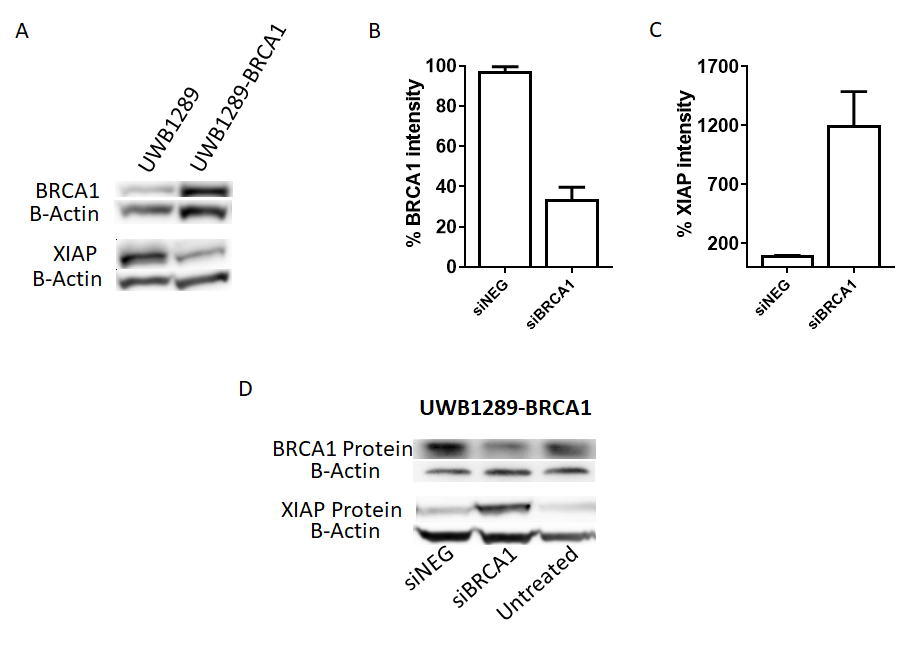


Fig.2 suppl. A) The level of expression of BRCA1 protein, evaluated by western blot, was higher in the *BRCA1*-restored OC CL UWB1289-BRCA1 compared with the *BRCA1*-mutated parental OC CL UWB1289-BRCA1. The level of expression of XIAP protein, evaluated by western blot, was lower in the *BRCA1*-restored OC CL UWB1289-BRCA1 compared with the *BRCA1*-mutated parental OC CL UWB1289-BRCA1.The gel bands have been juxtaposed for clarity. B) The level of expression of *BRCA1* mRNA, evaluated by qPCR, was lower after using siBRCA1 compared with the untreated *BRCA1*-restored OC CL UWB1289-BRCA1. There was no change in the expression after using a negative control (siNEG). C) The level of expression of *XIAP* mRNA, evaluated by qPCR, was higher after using siBRCA1 compared with the untreated *BRCA1*-restored OC CL UWB1289-BRCA1. There was no change in the expression after using a negative control (siNEG.). Note that in the figure, *BRCA1* and *XIAP* mRNA intensity is shown as a percentage of that in UWB1289-BRCA1 after normalization with the level of expression of GAPDH mRNA. D) The level of expression of BRCA1 protein in UWB1289-BRCA1, evaluated by western blot, was lower after treatment with siBRCA1 compared with the negative control (siNEG) and the untreated cell line. The level of expression of XIAP protein in UWB1289-BRCA1, evaluated by western blot, was higher after treatment with siBRCA1 compared with the negative control (siNEG) and the untreated cell line.The gel bands have been juxtaposed for clarity.


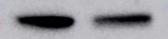

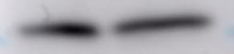

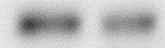

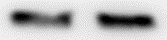


B-Actin

B-Actin

B-Actin

cIAP1


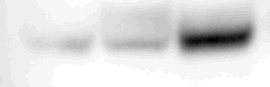

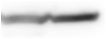


cIAP2

B-Actin

Survivin


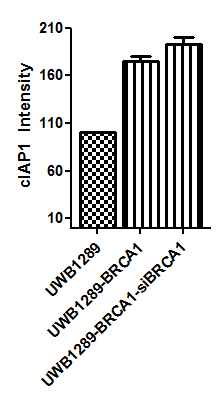


A)

B)

Livin

**UWB1289**

**UWB1289-BRCA1**


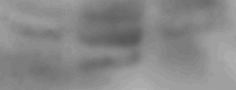

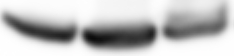


Fig.3 suppl. A) The level of expression of cIAP1 protein, evaluated by western blot, was higher in the *BRCA1*-restored OC CL UWB1289-BRCA1 compared with the *BRCA1*-mutated parental OC CL UWB1289. No differences in the levels of expression of cIAP2, Survivin or Livin proteins between UWB1289 and UWB1289-BRCA1 were observed. The gel bands have been juxtaposed for clarity. B) The level of expression of *cIAP1* mRNA, evaluated by qPCR, was higher in the *BRCA1*-restored OC CL UWB1289-BRCA1 compared with the *BRCA1*-mutated OC CL UWB1289. However, unlike XIAP, the silencing of wildtype *BRCA1*, using siRNA (siBRCA1), did not change *cIAP1* gene expression. Note that in the figure, cIAP mRNA intensity in the other CLs is shown as a percentage of that in UWB1289 after normalization with the level of expression of GAPDH mRNA in the corresponding CLs.


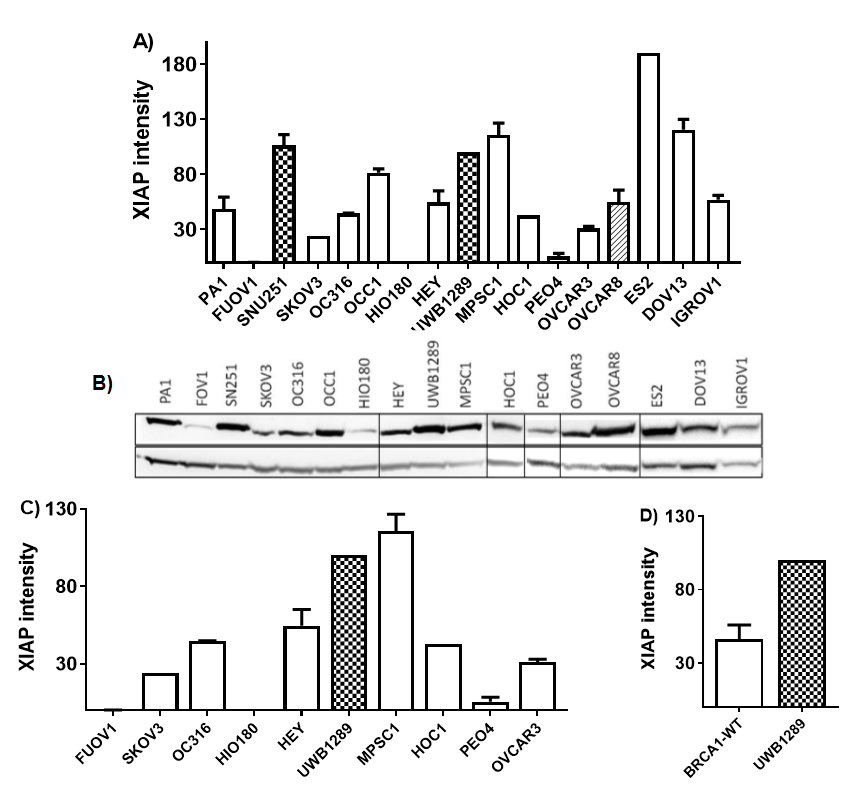


Fig.4 suppl. A-B) Evaluation by western blot of the level of expression of XIAP protein in a cell panel of 17 ovarian cancer (OC) cell lines (CLs). The gel bands have been juxtaposed from different gels for clarity. C) Evaluation by western blot of the level of expression of XIAP protein in a cell panel of 10 high grade serous OC CLs. D) *BRCA1*-mutated high grade OC CL shows a higher level of expression of XIAP protein compared with *BRCA1*-wildtype high grade OC CL. In this smaller subset the difference between these two groups is not statistically significant; however, the effect size remains strongly positive (p-value 0.24, Cohen's d = (100 - 45.86) ⁄ 30.907718 = 1.751666).

Note that in the figure, XIAP protein intensity in the CLs is calculated as a percentage of that in UWB1289 after normalization with the level of expression of GAPDH protein in the corresponding CLs. *BRCA1*-mutated OC CLs are highlighted with a pattern, the *BRCA1* methylated OC CL is highlighted with oblique banding and the BRCA1/BRCA2-wildtype OC CLs are highlighted in white.

HR=0.89 (CI=0.67-1.18) p-value=0.44

cIAP1 HIGH

B)

cIAP1 LOW

A)

Fig.5 suppl. A) In a group of 292 high grade serous OCs, cIAP1 levels, as determined by RPPA, are similar in OCs with *BRCA1* mutations (BRCA1-MUT) compared with OCs with wildtype *BRCA1/2* (BRCA1/2-WT) (p-value=0.9). cIAP1 protein intensity values in OCs were quantified by RPPA as described in the material and methods section and the values were visualized in the graph using Tukey boxplots. B) In a group of 422 OCs from patients treated with surgery and adjuvant platinum-based chemotherapy, those patients with cancers expressing a level of cIAP1 protein higher than the median had the same overall survival than patients with cancers expressing a level of cIAP1 protein lower than the median.


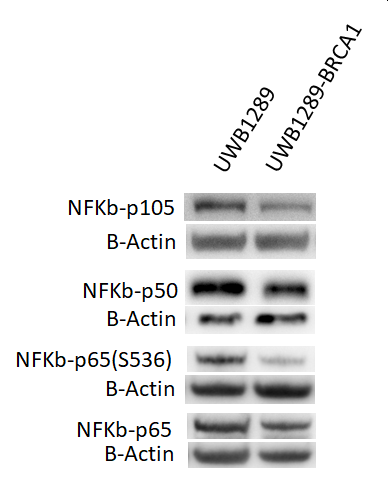


Fig.6 suppl. The level of expression of NFKB-p105, NFKB-p50, NFKB-p65(S536) proteins, evaluated by western blot, were higher in the *BRCA1*-mutated parental OC CL UWB1289 compared with the *BRCA1*-restored OC CL UWB1289-BRCA1. The gel bands have been juxtaposed for clarity.

B)

A)

Fig.7 suppl. A) Effect on cell growth by the inhibitor of NF-kB pathway BMS-345541 in a panel of 17 ovarian cancer cell lines. B) *BRCA1*-mutated ovarian cancer cells lines (BRCA1-MUT) are on average more significantly sensitive to growth inhibition by BMS-345541 than *BRCA1*-wildtype (BRCA1-WT) OC CLs (p-value= 0.007). In the bar chart *BRCA1*-mutated OC CLs are highlighted with a pattern, the *BRCA1*-methylated OC CL is highlighted with oblique banding, and BRCA1-wildtype OC CLs are highlighted in white.


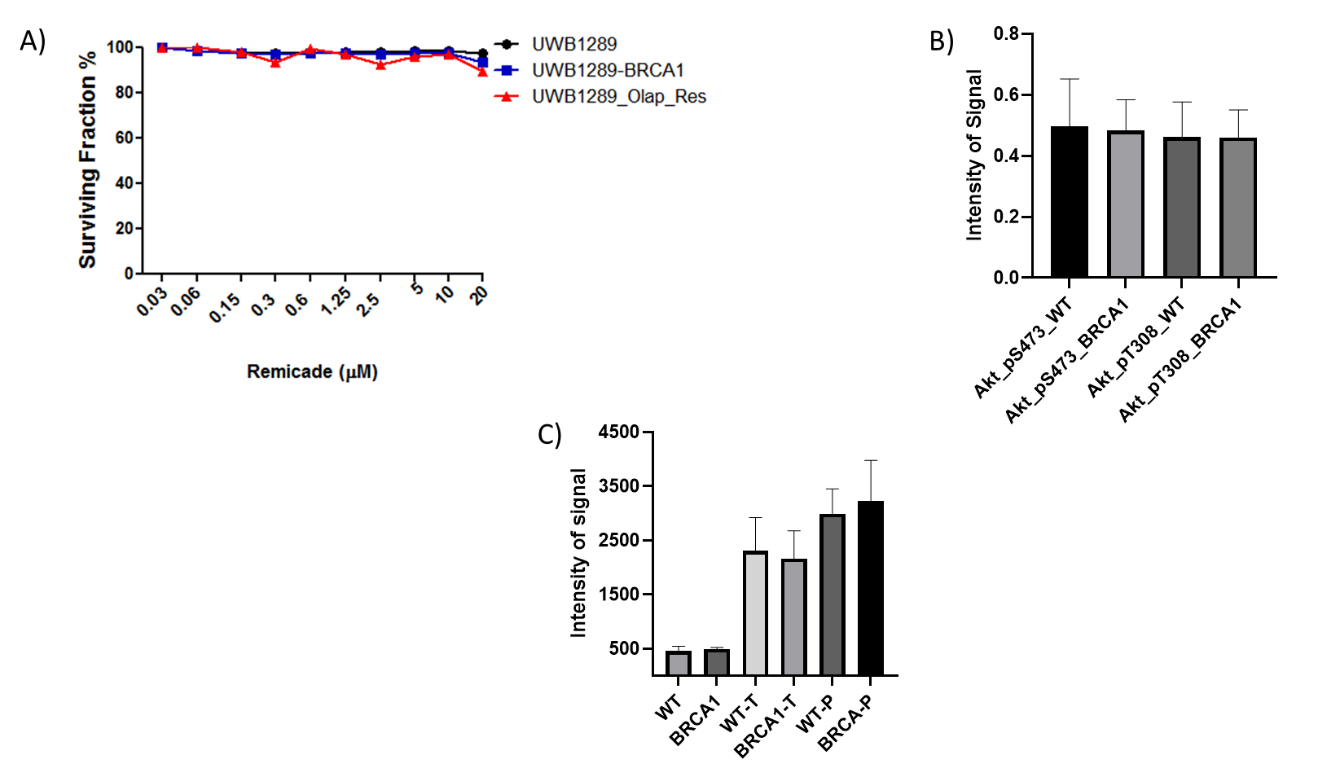


Fig.8 suppl. A) Evaluation of the effect of Inflximab (Ramicade) on the *BRCA1*-mutated ovarian cancer cell line UWB1289 and the *BRCA1*-mutated ovarian cancer cell line with acquired resistance to olaparib (n=3) UWB1289-RES. B) The level of expression of AKT(S473) and AKT(T308) protein, evaluated by RPPA, in *BRCA1*-mutated ovarian cancer cell line (_WT) UWB1289 compared with the *BRCA1*-restored OC CL UWB1289-BRCA1 (_BRCA1) (n=3) C) Evaluation of the ROS generation in *BRCA1*-mutated ovarian cancer cell line (_WT) UWB1289 compared with the *BRCA1*-restored OC CL UWB1289-BRCA1 (_BRCA1) (n=3) by DCFDA/H2DCFDA – Cellular Ros assay kit (ab113851). WT and BRCA1 untreated cells. WT-T and BRCA1-T cells treated with H_2_O_2_ for 30 min. WT-T and BRCA1-T cells treated with H_2_O_2_ for 30 min. WT-P and BRCA1-P cells treated with TBHP for 30 min as positive control.


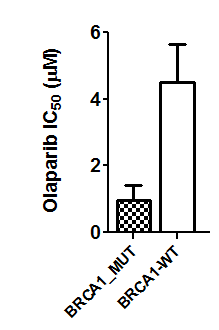

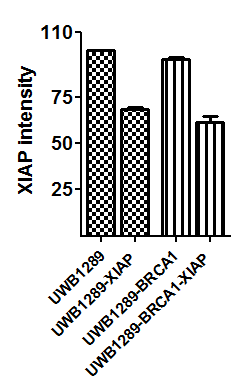

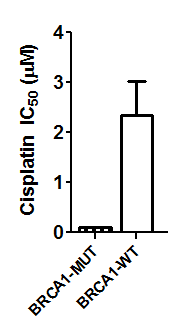


A)

C)

B)

p-value=0.012

p-value=0.01

p-value=0.003

p-value=0.005

Fig.9 suppl. A) *BRCA1*-mutated OC CLs (BRCA1-MUT) are on average more sensitive to cisplatin than *BRCA1*-wildtype (BRCA1-WT) OC CLs. B) *BRCA1*-mutated OC CLs (BRCA1-MUT) are on average more sensitive to olaparib (PARP inhibitor) than *BRCA1*-wildtype (BRCA1-WT) OC CLs. C) The treatment with 25nM XIAP siRNA for 24hrs led to a decrease of XIAP protein expression in both UWB1289 and UWB1289-BRCA1 in comparison with control-treated cells. Note that in the figure, XIAP protein intensity in the CLs is shown as a percentage of that in control-treated UWB1289 after normalization with the level of expression of GAPDH mRNA in the corresponding CLs. *BRCA1*-mutated OC CLs are highlighted with a pattern, the *BRCA1*-methylated OC CL is highlighted with oblique banding, the *BRCA1*-restored OC CL is highlighted with horizontal banding and BRCA1-wildtype OC CLs are highlighted in white.


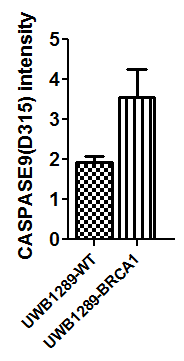

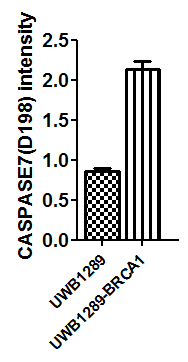


A)


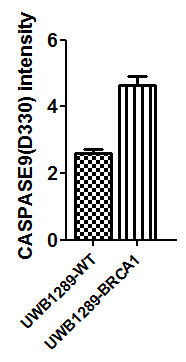


B)

C)

p-value=0.011

p-value=0.042

p-value=0.018

Fig.10 suppl A/B/C) The level of cleaved (activated) caspases, evaluated by RPPA, was lower in the *BRCA1*-mutated ovarian cancer cell line UWB1289 compared with the *BRCA1*-restored OC CL UWB1289-BRCA1. The caspase intensity values in the OCs was quantified by RPPA as reported in the material and methods section. Note that *BRCA1*-mutated OC CLs are highlighted with a pattern, the *BRCA1*-restored OC CL is highlighted with horizontal banding.

A)

B)

Fig.11 suppl. A) Treatment with 0.5 µM BV6 for 72 hours led to an inhibition of ≤20% in the proliferation ovarian cancer cell lines (CLs). These data were normalized to the proliferation of the same control treated CLs. B) After 72 hours of treatment with BV6, the levels of XIAP protein, as evaluated by RPPA were decreased in all cell lines as expected. XIAP intensity values in the OCs CL was quantified by RPPA as reported in the materials and methods section. Note that in the figure *BRCA1*-mutated OC CLs are highlighted with a pattern, the *BRCA1*-restored OC CL is highlighted with horizontal banding.


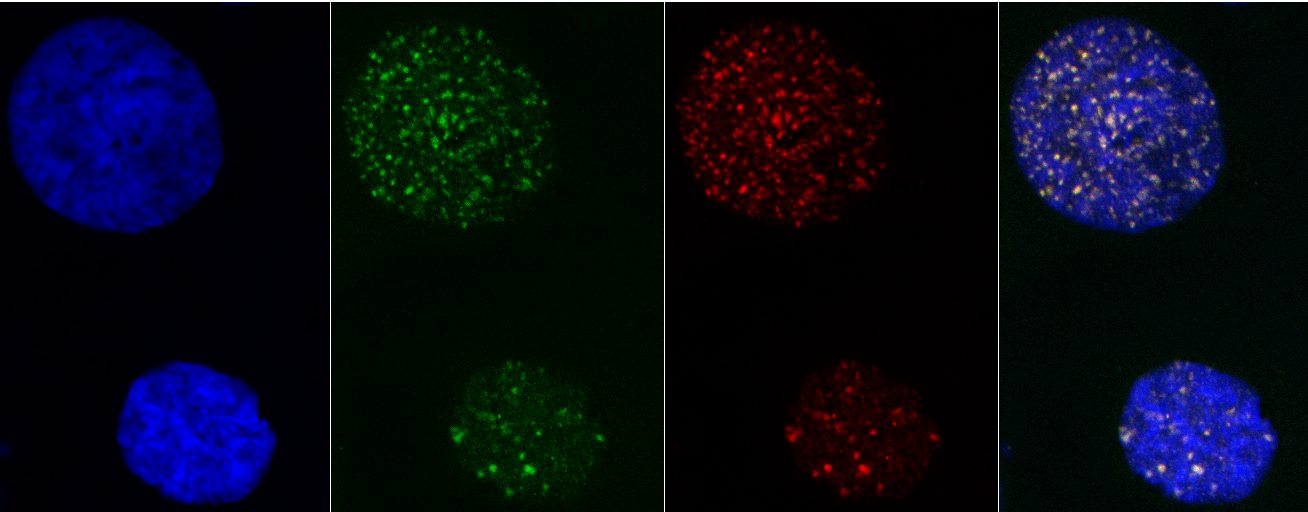


Olap 0.5 uM

DAPI

H2AX

RAD51

Co-localization


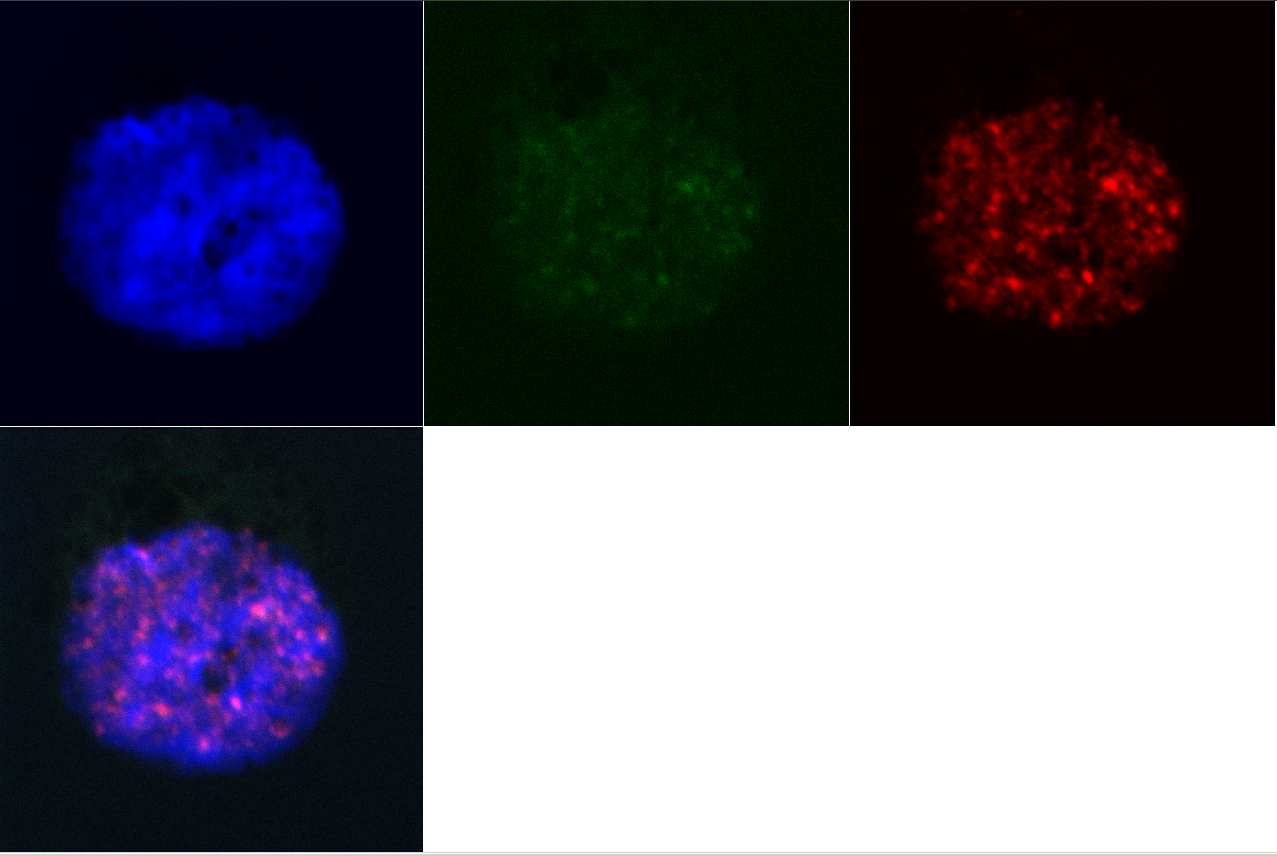

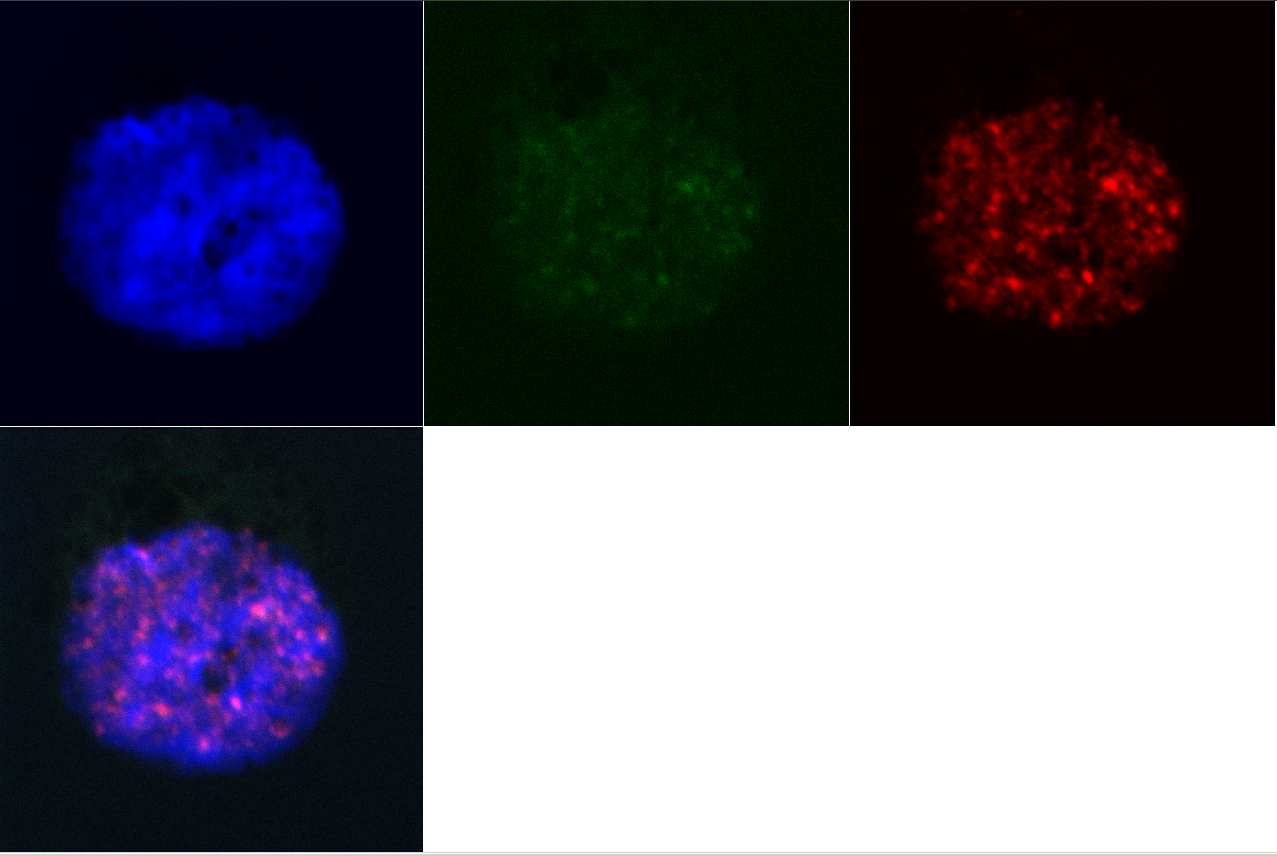


Olap 0.5 uM

Pre-treated

LCL161

A

B

Fig.12 suppl. A) The acquired resistance to Olaparib in the UWB1289-RES results in restoration of homologous recombination (HR) competence. HR competence was evaluated using an HR assay (see Fig.1 suppl. for details) after a treatment with Olaparib for 48 hrs. B) A pre-treatment with IAP inhibitor LCL161 (for 72 hrs) impairs the HR competence in the cell UWB1289-RES.

|  | **Dataset A** | | **Dataset B** | | **Dataset C** | |
| --- | --- | --- | --- | --- | --- | --- |
|  | **Number** | **%** | **Number** | **%** | **Number** | **%** |
| **Total Patients** | 69 |  | 292 |  | 130 |  |
| **Age at diagnosis, years** |  |  |  |  |  |  |
| **Range** | 35-84 |  | 26-89 |  | 31-84 |  |
| **Median** | 56 |  | 59 |  | 58 |  |
| **Follow-up, months** |  |  |  |  |  |  |
| **Range** | 3-71 |  | 1-182 |  | 1-128 |  |
| **Median** | 17 |  | 27 |  | 45 |  |
| **Stage at dignosis** |  |  |  |  |  |  |
| **1** | 6 | 9 | 13 | 4 | 0 | 0 |
| **2** | 5 | 7 | 15 | 5 | 0 | 0 |
| **3** | 49 | 71 | 228 | 78 | 104 | 80 |
| **4** | 9 | 13 | 34 | 12 | 26 | 20 |
| **Unknown** | 0 | 0 | 2 | 1 | 0 | 0 |
| **Histology** |  |  |  |  |  |  |
| **Serous** | 69 | 100 | 292 | 100 | 130 | 100 |
| **Unknown** | 0 | 0 | 0 | 0 | 0 | 0 |
| **Grade** |  |  |  |  |  |  |
| **1** | 3 | 4 | 0 | 0 | 0 | 0 |
| **2** | 11 | 16 | 31 | 10 | 63 | 48 |
| **3** | 55 | 80 | 203 | 70 | 67 | 52 |
| **4** | 0 | 0 | 0 | 0 | 0 | 0 |
| **Unknown** | 0 | 0 | 58 | 20 | 0 | 0 |
| **Surgery** |  |  |  |  |  |  |
| **Yes** | 69 | 100 | 208 | 71 | 130 | 100 |
| **No** | 0 | 0 | 0 | 0 | 0 | 0 |
| **Unknown** | 0 | 0 | 84 | 28 | 0 | 0 |
| **Residual after surgery, cm** |  |  |  |  |  |  |
| **0** | 9 | 13 | 41 | 14 | 13 | 10 |
| **<1** | 2 | 3 | 101 | 35 | 13 | 10 |
| **>1** | 57 | 83 | 66 | 22 | 13 | 10 |
| **Unknown** | 1 | 1 | 84 | 29 | 91 | 70 |
| **Chemotherapy** |  |  |  |  |  |  |
| **Yes** | 69 | 100 | 208 | 71 | 130 | 100 |
| **No** | 0 | 0 | 0 | 0 | 0 | 0 |
| **Unknown** | 0 | 0 | 84 | 28 | 0 | 0 |

Table 1 suppl. Patient Characteristics.


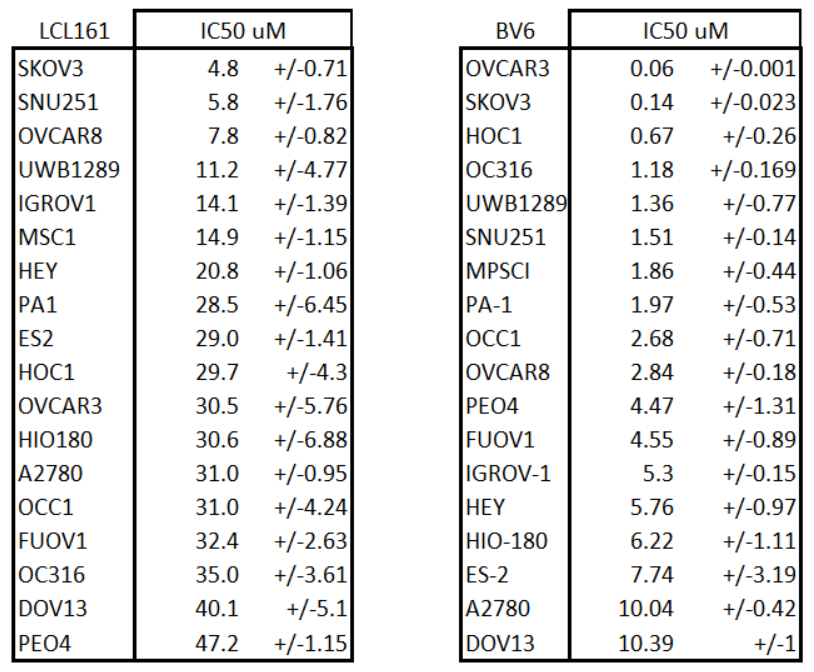


Table 2 suppl. BV6 and LCL161 IC_50_s in a panel of 18 ovarian cancer cell lines.

| Patient ID | Patient age | Diagnosis | Previous treatment | *BRCA1/2* status | Other known molecular features of the PDX | PDX cisplatin response |
| --- | --- | --- | --- | --- | --- | --- |
| #13 | 51-60 | HGSOC  Stage IIIC | Chemo-naïve | *gBRCA2* mutation | *TP53* mutation; *MAP2K1* mutation | Resistant |
| #56 | 31-40 | HGSOC  Stage IIIC | Chemo-naïve | *gBRCA1*  mutation | *TP53* mutation; *RB1* truncation | Sensitive |
| #62 | 61-70 | HGS FT  Stage IIC | Chemo-naïve | *BRCA1* methylation  (homozygous) | *TP53* mutation; *FGF12*, *NFKBIA, NKX2-1* and *ZNF217* amplifications; *CTNNA1* loss; *EZH2* truncation | Refractory |
| #201 | 61-70 | HGSOC  Stage IIIC | Chemo-naïve | Wild type | *TP53* mutation; *MDM2, ARFRP1, CCNE1, FRS2, TOP1, AURKA, SRC, MYC, ZNF217, BCL2L1, FGF10, RICTOR* and *GNAS* amplifications; *NOTCH3* mutation; *PTEN* deletion | Refractory |
| #931 | 51-60 | HGSOC  Stage IV | Post-7 lines of chemo or bevacizumab  (no PARPi) | *gBRCA2*  mutation  with 2°  reversion mutation | *TP53* mutation; *SOX2, CCNE1, BCL2L1, PIK3CA, PRKCI* and *TERC* amplifications; *RB1* deletion | Refractory |

Table 3 suppl: Summary characteristics of the PDX models used in this study.

PDX #13, #56 and #62 were first described by Topp et al^22^ and PDX #201 by Kondrashova et al^23^. As previously published, the PDX models were molecularly characterised using Foundation Medicine’s FoundationOne T5a panel, BROCA assay and *BRCA1* promoter methylation testing.

PDX cisplatin response has been previously defined: sensitive, prolonged response with no progressive disease (PD) before 100 days; resistant, initial tumour response/regression followed by PD observed before 100 days; refractory, continues to grow on treatment or has a time to PD of less than 50 days^22^.

| Cell line | Original tumor histology | Isolated from | Pre‐isolation | | Reported mutations | References |
| --- | --- | --- | --- | --- | --- | --- |
|  |  |  | Treatment received | Response |  |  |
| DOV13 | Adenocarcinoma | Unknown | Unknown | Unknown | TP53 | (Young et al., 1995) |
| ES2 | clear cell | Primary tumour | None | N/A | TP53, STAG2 | (2012b) (Sikic, 2012) |
| FUOV1 | Serous | Primary tumour | None | N/A | TP53, | (Emoto et al., 1999) |
| HEY | Serous | Peritoneal deposit and xenograft | Radiotherapy, radium | CR | BRAF, KRAS | (Buick et al., 1985; Hills et al., 1989) |
| HI0180 | Normal OSE | Normal OSE | N/A | N/A |  | (Sood et al., 2002) |
| HOC1 | Serous | Ascites | MEL, CIS, ADR, CYC | PR, PR |  | (Buick et al., 1985; Mackillop et al., 1983) |
| IGROV1 | Endometriod/clear cell | Primary tumour | None | N/A | PIK3CA , PTEN, TP53 | (Benard et al., 1985) |
| MPSCI | Serous | Unknown | Unknown | Unknown |  | (Pohl et al., 2005) |
| OC316 | Serous | Ascites | CIS, ETO, CYC, TAX | PD, SD | TP53 | (Alama et al., 1996) |
| OCC1 | Clear cell | Ascites | Unknown | Unknown |  | (Wong et al., 1990) |
| OVCAR3 | Serous | Ascites | CYC, CIS, DOX | Unknown | PIK3R1, TP53 | (Hamilton et al., 1983; Hills et al., 1989; Schilder et al., 1990) |
| OVCAR8 | Adenocarcinoma | Unknown | CAR | PD | TP53, | (Schilder et al., 1990) |
| PA1 | Germ cell tumor | Ascites | Chemotherapy | NR | TP53, | (Hills et al., 1989) |
| PEO4 | Serous | Ascites | CIS, CHL, 5‐FU | CR | TP53, NF1 | (Langdon et al., 1988; Sakai et al., 2009) |
| SKOV3 | Serous | Ascites | THI | Unknown | PI3KCA, TP53 | (Hills et al., 1989) |
| SNU251 | Endometriod | Ascites | CYC, ADR, CIS | Unknown | BRCA1 | (Yuan et al., 1997) |
| UWB1.289 | Serous | Primary tumour | TAX, CAR, TAP | PR | BRCA1 | (DelloRusso et al.,2007) |

ADR – Adriamycin, CAR – Carboplatin, CIS – Cisplatin, CHL – Chlorambucil, CR – Complete Response, CYC – Cyclophosphamide, DOX – Doxurubicin, ETO – Etoposide, MEL – Melphalan, N/A – Not Applicable, NR – No Response, OSE – Ovarian Surface Epithelium, PD – Progressive Disese, PR – Partial Response, TAP –Tapotecan, TAX – Taxol, THI – Thiotepa.

Table 4 suppl. Clinical characteristics of ovarian tumors from which cell lines were established.

**Cell line panel and cell culture - Supplementary details**

The *BRCA1*-mutated cell line UWB1289 and UWB1289-BRCA1, a stable cell line derived by restoration of wild-type *BRCA1* to UWB1289, were purchased directly from the ATCC (#CRL-2945 and #CRL-2946 respectively). All other cell lines were obtained from M.D. Anderson Cancer Center (USA) and authenticated by Source BioScience LifeSciences (UK) using the AmpFISTR® SGM Plus® PCR amplification kit on the following dates:

SNU251-04/04/2013

OVCAR8-30/09/2013

SKOV3-04/04/2013

HOC1-14/9/2013

OC316-22/03/2013

MPSCI-04/04/2013

PA1-04/04/2013

OCC-1-04/042013

PEO4-04/04/2013

FUOV1-04/04/2013

IGROV1-05/06/2012

HEY-30/07/2012

HIO180-04/04/2013

ES-2-04/04/2013

POV13-30/07/2012

After their authentication the cell lines were expanded and aliquots made. Each aliquot was maintained in the laboratory for a maximum of 15 passages after resuscitation before being discarded and replaced.

**Supplementary Material and Methods**

**Reverse Phase Protein Array**

Protein extraction

Lysis buffer was prepared as follows, 150 Mm sodium chloride, 50 mM HEPES pH 7.4, 1.5 mM magnesium chloride, 1 mM EGTA, 100 mM NAF, 10 Mm sodium pyrophosphate, 1 mM sodium orthovanadate, %1 triton 100-x, 10% glycerol.

After incubation on ice for 15 min, samples were vortexed and centrifuged at 15000 rpm at +4 °C for 15 min. Protein extraction lysates were normalised to a 1.5 μg/μL as assessed by a bicinchoninic acid assay (DC Protein Assay—Bio-Rad, California, USA).

Three parts of cell lysates were mixed with one part SDS buffer (40% Glycerol, 8% SDS, 0.25 M Tris-HCL, pH 6.8 plus Bond-Breaker TCEP Solution (Pierce Biotechnology, Illinois, USA) at 1/10th of the volume) and boiled. Lysates were manually diluted in fourfold serial dilutions with lysis buffer. A sample arrays have been created on Oncyte Avid nitrocellulose-coated slides (Grace Bio-Labs, Oregon, USA) by an Aushon 2470 arrayer (Quanterix, Billerica,MA) per manufacturer’s protocol.

The slides were store at −80°C prior to immunostaining. Immunostaining was performed on an automated slide stainer (Dako Link 48—Dako, California, USA) according to the manufacturer's instructions (CSA kit—Dako).

Each slide was incubated with a single primary antibody (see online supplementary table S3) at room temperature for 30 min. All antibodies used were listed in the Standard Antibody List 298 and validated by the MD Anderson Cancer Center as well as internally.
Secondary antibodies were goat anti-rabbit IgG (1:5000) (Vector Laboratories, California, USA) or rabbit anti-mouse IgG (1:10) (Dako). Dako secondary antibodies were used as a starting point for amplification via horseradish peroxidase-mediated biotinyl tyramide with chromogenic detection (diaminobenzidine) according to the manufacturer's instructions (Dako).

Scanned TIFF images of slides were analysed using MicroVigene software V.5.1 (VigeneTech, Massachusetts, USA) to generate spot signal intensities.13 Instead of generating multiple linear regression curves for data quantification over each series of serial dilutions, the RPPA module of MicroVigene uses a four-parameter logistic-log model (‘SuperCurve’ algorithm) with all spots within one array employed to form a sigmoid antigen-binding kinetic curve. The protein concentrations were normalized by global sample median normalization.

**Mutation Screening**

PCR was performed on 2 ng of DNA in a 3-μL reaction using the primers flanking the exons of *BRCA½* that are used in the BRCAnalysis (Myriad Genetics, Salt Lake City, UT) clinical test with the following cycling conditions: 95°C × 10 minutes, 35 cycles of 95°C × 30 seconds, 62°C × 30 seconds, and 72°C × 1 minute, finishing with 72°C × 1 minute. Each PCR product was treated with 0.1 U of Shrimp Alkaline Phosphatase (Sigma-Aldrich, St Louis, MO.) The PCR product was diluted 1:9, and 0.8 μL was used for cycle sequencing with Big Dye Sequencing Chemistry and Taq FS (Applied Biosystems). Cycle conditions were 95°C × 3 minutes, 32 cycles of 95°C × 30 seconds, 50°C × 30 seconds, 60°C × 3 minutes, and 72°C × 10 minutes. Sequence products were run on a Megabace 4500 automated sequencer (GE Medical Systems, Milwaukee, WI) per manufacturer's protocol.

### **BRCA1 promoter methylation qPCR assays**

The Methyl-Profiler DNA Methylation PCR Array System (SA BioSciences) was used to quantify methylation levels following the manufacturers recommended protocol. DNA methylation-sensitive and methylation-dependent restriction enzymes were used to selectively digest unmethylated or methylated genomic DNA, respectively. Post-digest DNA was quantified by real-time PCR using primers flanking the regions of interest, BRCA1 (MePH28472-1A). The relative concentrations of differentially methylated DNA are determined by comparing the amount of each digest with that of a mock digest.

### **Cytotoxicity-proliferation assays**

To determine the resistance to chemotherapy drugs, cells were plated into flat-bottomed, 96-well plates at the cell density shown of 1 × 10^3^ cells/well and allowed to attach overnight. Slower growing cells (SNU-251) were plated at 2 × 10^3^ cells/well. Olaparib (AZD2281) and veliparib (ABT888) were purchased from Selleck Chemicals (Boston, MA, USA) and made up in DMSO. Wells were treated in triplicate with serial dilutions of drug in a final volume of 200 μL. Drug-free controls were included in each assay. DMSO controls were also performed for each cell line. Plates were incubated for a further 5 days at 37 °C in a humidified atmosphere with 5% CO_2_ and cell viability was determined using an acid phosphatase assay[^1^](#_ENREF_1)

1 Martin, A. & Clynes, M. Comparison of 5 microplate colorimetric assays for in vitro cytotoxicity testing and cell proliferation assays. *Cytotechnology* **11**, 49-58 (1993).

**Chromatin Immunoprecipitation Assay**

Growing cells (3 x 10^7^) were treated with formaldehyde to cross-link DNA and associated proteins. Cross-linked chromatin was extracted, sheared by sonication, and incubated with antibody overnight at 4°C and subsequently incubated with protein A-agarose beads.

After washing, immune complexes were eluted from the beads, heated to reverse the cross-links, and treated with proteinase K and RNase A to remove proteins and any contaminating RNA. DNA was analyzed by PCR using primers that generate a 106-bp product that corresponds to a region (-271 to -375) of the human XIAP promoter.

forward primer, 5’-TGCCTGCTTAAATATTACTTTCCTCAAAA-3’

reverse primer, 5’-ACTACACGACCGCTAAGAAACATTCT-3’.

As a negative control, PCR reactions were performed using primers specific for the human actin promoter. The actin sequences used were as follows:

forward primer, 5’-TGCCTAGGTCACCCACTAACG-3’;

reverse primer, 5’-CTGGAGCTGCCTGCTTTTG-3’

PCR products were detected on 2.5% agarose gels.
